# Incidence, persistence, and clearance of anogenital human papillomavirus among men who have sex with men in Taiwan: a community cohort study

**DOI:** 10.3389/fimmu.2023.1190007

**Published:** 2023-06-20

**Authors:** Xinyi Zhou, Tian Tian, Zhen Lu, Yi-Fang Yu, Yuwei Li, Yiguo Zhou, Yi-Fan Lin, Carol Strong, Huachun Zou

**Affiliations:** ^1^ School of Public Health (Shenzhen), Sun Yat‐sen University, Shenzhen, China; ^2^ Department of Public Health, College of Medicine, National Cheng Kung University, Tainan, Taiwan; ^3^ School of Public Health, Peking University, Beijing, China; ^4^ Kirby Institute, University of New South Wales, Sydney, Australia

**Keywords:** human papillomavirus, incidence, persistence, clearance, men who have men with men

## Abstract

**Background:**

Men who have sex with men (MSM) have an increased risk of human papillomavirus (HPV) infection. This study aimed to assess the incidence, persistence, and clearance of anogenital HPV infections among MSM and the correlates in a 3-year community cohort study.

**Methods:**

From 2015 to 2019, MSM were recruited and followed up at 6, 12, 24, and 36 months in Taiwan. Questionnaires and anogenital swabs were collected at baseline and each follow-up visit. Thirty-seven HPV genotypes were tested and genotyped using the linear array HPV genotyping test. The incidence, persistence, and clearance rates of anogenital HPV infection and 95% confidence intervals (CIs) were estimated through Poisson regression. Correlates of the incidence and clearance rates were examined using a generalized estimating equations (GEE) model.

**Results:**

A total of 201 MSM were retained in the cohort study with a median age of 27 years (interquartile range [IQR]: 24–32) at baseline. The incidence, persistence, and clearance rates of any anal HPV infection among MSM were 43.6 (95% CI: 33.7–55.6), 23.4 (17.7–30.2), and 58.3 (45.1–74.1) per 1,000 person months (pms), respectively. The incidence, persistence, and clearance rates of any penile HPV infection among MSM were 26.8 (20.1–34.9), 13.4 (8.0–20.9), and 51.5 (37.8–68.5) pms, respectively. MSM who did not consistently use a condom in receptive sex (adjusted odds ratio [AOR]: 2.06, 95% CIs: 1.14–3.72) were more likely to acquire any anal HPV infection. Age at recruitment (1.05, 1.01–1.09) was positively associated with any penile HPV incidence. MSM with over one sex partner in receptive anal sex (0.53, 0.30–0.94) were less likely to clear any anal HPV infection. MSM who were unemployed/students (0.55, 0.30–0.98) were less likely to clear any penile HPV infection.

**Conclusion:**

High incidence and low clearance of anogenital HPV infection among MSM in the study serve as a reminder that this population needs to be targeted for HPV vaccination. It is essential for MSM to scale up HPV screening and adhere to safe sex.

## Introduction

Human papillomavirus (HPV) infections can occur at multiple anatomic sites, such as the oral cavity, pharynx, anus, and penis (1). Persistent HPV infections could give rise to diseases like genital warts, penile cancer, anal cancer, and oropharyngeal cancer in men (2–5). Worldwide, 690,000 new cancer cases were attributable to HPV infection in 2018, with an age-standardized incidence rate of 8.0 cases per 100,000 person-years (6). In the United States, HPV is thought to be responsible for approximately 90% of anal cancers, 60% of penile cancers, and 70% of oropharynx cancers (7). Men who have sex with men (MSM) are at a higher risk for anogenital HPV infections than heterosexual men (8, 9).

Previous studies have investigated the natural history of anal HPV infection among MSM. Dona and colleagues reported that the incidence and clearance rates of anal HPV infections for any genotype were 85.6 and 35.6 per 1,000 person months (pms) (10). In mainland China, Zhang et al. reported that the incidence and clearance rates of anal HPV for any genotype were 53.4 and 50.9/1,000 pms (11). Zhou et al. investigated the natural history of HPV infection, including persistence rates (12). These studies with a continuous concern focus on HPV infection at the anus, but a few publications have reported on the natural history of penile HPV in recent years. A study reported the natural history of genital HPV infection in men (with 122 MSM) in 2011 (13). A Dutch study investigated penile HPV infection among 445 MSM recruited over the last decade (14). Therefore, the natural history of penile HPV infection needs more timely reporting in China due to the increasing age-standardized incidence found in the country (15). Moreover, only a French study with 150 samples (2016–2018) simultaneously assessed the natural history of HPV at the anus and penis (16). Articles about the natural history of HPV infection at both the anus and penis among MSM in China have been few.

Several countries, mostly developed ones, have introduced gender-neutral HPV vaccination programs, such as Australia, the US, and the UK (17). Although HPV vaccines have been demonstrated to be protective against HPV infections and related diseases in men (18), China’s HPV vaccination programs exclude men in mainland regions nonetheless. Recently, men aged 9–26 and 9–45 can voluntarily receive quadrivalent (4V) and 9-valent (9V) HPV vaccines out of pocket in Taiwan. The optimization and implementation of the aforementioned HPV vaccination programs need to be addressed and promoted through more research evidence regarding anogenital HPV infection under different political, economic, and medical settings.

To our knowledge, no previous studies have reported the incidence, persistence, and clearance of anogenital HPV among MSM in Taiwan. This study aimed to investigate the natural history of HPV infections at the anus and penis. Additionally, we assessed factors associated with the incidence and clearance of any anogenital HPV infections. The evidence from our investigation could help promote the launch of HPV vaccination in MSM populations in mainland China and Taiwan.

## Materials and Methods

### Study population

A community cohort study was conducted among MSM between October 2015 and June 2019 in Taiwan. Recruitment from community health centers that provide HIV testing and consultation services was carried out on social media. This observational study focused on young MSM and had a follow-up duration of 3 years. The study was conducted within the community setting, which enabled us to effectively manage on-site investigations, ensuring consistency in survey administration, sample collection, and testing procedures. Eligible participants had previously had sex (including mutual masturbation, oral sex, or anal sex) with another man, and were willing to participate in the cohort study. All participants provided written informed consent. The study was approved by the Ethics Committee of the National Cheng Kung University Hospital (reference number: A-BR-103-075).

A total of 253 MSM were recruited at baseline between October 2015 and May 2016. Socio-demographic characteristics, sexual behaviors, and other information were collected at baseline. Anal and penile HPV testing for participants was provided at baseline and each follow-up at 6, 12, 24, and 36 months. Details about the study recruitment have been described previously (19–21). Participants who completed the questionnaire and had taken part in at least one follow-up were included in the present study. In this community cohort study, we implemented several measures to ensure quality control. First, data quality control measures were employed, including timely checks after data collection and during data collation. These checks assessed the credibility and logic of the generated questionnaire data, such as the questionnaire number, data missing and errors, consistent rate of repeated questions, and correctness of logical conditions. Second, we ensured the quality of biological sample collection, processing, and preservation. The laboratory apparatus and supplies used in the company were authenticated to meet the required standards for sample processing and preservation. Last, we provided training and assessment for investigators involved in the study. Regular site visits by supervisors were conducted to monitor work progress and standardize the workflow, ensuring adherence to the predetermined procedures.

### HPV detection and genotyping

HPV DNA testing and genotyping were performed using the linear array HPV genotyping test (Roche Molecular Diagnostics, Pleasanton, CA, USA) with good type-specific reproducibility and reliability to identify 37 HPV genotypes (22), including 21 high-risk (16, 18, 26, 31, 33, 35, 39, 45, 51, 52, 53, 56, 58, 59, 66, 67, 68, 69, 70, 73 [MM9], 82 [MM4]) and 16 low-risk HPV genotypes (6, 11, 40, 42, 54, 55, 61, 62, 64, 71 [CP8061], 72, 81 [CP8304], 83 [MM7], 84 [MM8], 82v, 89). Details about HPV specimen collection and detection procedures have been reported previously (20, 21).

### Statistical analyses

Rates of incidence, persistence, and clearance of HPV infection at both anal and penile sites were calculated for any genotype, high-risk genotype, low-risk genotype, 9V genotype (6, 11, 16, 18, 31, 33, 45, 52, 58), 4V genotype (6, 11, 16, 18), HPV 16/18, HPV 6/11, and individual HPV genotype. Any genotype was defined as a positive test result for at least one of 37 HPV genotypes. Positive results with single or multiple grouped (high risk, low-risk, 9V, and 4V) HPV genotypes were defined as relevant grouped genotypes.

As the numerator, incident HPV infection was defined as the first occurrence of a positive test result at any follow-up after a negative test result for the same grouped or individual genotype (0–1). As the denominator of incidence rate, pms were calculated as the time from the date of the visit with a negative HPV test result to the date of the first positive test result or to the last visit if the test remained negative. Persistent infection was defined as positive test results for the same grouped or individual HPV genotype detected at two successive tests (1–1). We estimated pm as the time from the visit date of a positive HPV test result to the date of the same positive test result. Clearance of HPV infection was defined as the first negative test result at any follow-up after a positive test result for the same grouped or individual genotype (1–0). We calculated pm of clearance rate as the time from the visit date of a positive HPV test result to the date of the first negative test result or to the last visit if the test remained positive. Incidence, persistence, and clearance rates were expressed as events per 1,000 pms. Corresponding 95% confidence intervals (CIs) were calculated using Poisson regression.

Attrition analyses were used to examine the differences in baseline demographic characteristics and sexual behaviors between the retained and baseline participants using Fisher’s exact tests. Additionally, the incidence rates for any, high-risk, and low-risk anogenital HPV infection were compared. We applied generalized estimating equations (GEE) logistic regression with robust standard errors and an exchangeable correlation structure to evaluate factors associated with the incidence and clearance of any HPV infection. The GEE method is applicable to longitudinal data with relative robustness, and it is suitable for situations with limited data (smaller sample sizes) availability (23). Variables with a *P*<0.20 in univariate analyses were included in multivariable regression to estimate the odds ratios (OR), adjusted odds ratios (AOR), and 95% CIs. Rates of HPV incidence, clearance, and persistence were calculated across age groups. Age was divided into four groups (≤24 years, 25–27 years, 28–32 years, and >32 years) using cutoff points close to quartiles. All statistical analyses were performed using R 4.1.1 (R Core Team, Vienna, Austria).

## Results

### Population characteristics

We excluded men with missing information, and those who never participated in the follow-up were not included in the cohort. A total of 201 (82%) MSM were retained in the entire cohort. The median age of enrolled MSM was 27 years (interquartile range [IQR]: 24–32) (**Supplementary file**, **Table S1**). At the baseline, most of the participants were unmarried (56.2%), employed (65.3%), homosexual (80.8%), had a monthly income <40,000 NT (75.2%), had never had an HPV test (92.0%), and had not had an STI diagnosis in the past year (78.4%). The estimated attrition rates of 6-, 12-, 24-, and 36-month follow-up were 26.5%, 36.0%, 49.0%, and 59.3% respectively. We found statistically significant differences between the entire cohort and lost ones for “No. of insertive anal sex partners in the past year” (*P*=0.021) (**Supplementary file**, **Table S1**). There were no significant differences between the groups in the incidence rates of any, high-risk, and low-risk HPV infection (**Supplementary file**, **Table S2**). According to the attrition analyses, we conducted further statistical analyses with these variables and remained prudent with our interpretation.

### Incidence, persistence, and clearance of HPV among MSM

Rates of incidence, persistence, and clearance are shown in **Tables 1** and **2**. Among 201 MSM who were enrolled in the cohort study, 70 and 29 had anal and penile HPV infection, respectively, with any genotype at baseline.

**Table 1:** Incidence/persistence/clearance of anal HPV infection among men who have sex with men.

HPV genotypes	Incidence			Persistence			Clearance		
	No.	Person Months	Rate (95%CI) per 1000 Person Months	No.	Person Months	Rate (95%CI) per 1000 Person Months	No.	Person Months	Rate (95%CI) per 1000 Person Months
Any genotype^a^	65	1490	43.6 (33.7-55.6)	58	2483	23.4 (17.7-30.2)	66	1133	58.3 (45.1-74.1)
HR genotypes^b^									
** Any HR genotype**	47	2018	23.3 (17.1-31.0)	39	1895	20.6 (14.6-28.1)	51	927	55.0 (41.0-72.3)
** HPV 16**	7	2483	2.8 (1.1-5.8)	3	251	12.0 (2.5-34.9)	9	139	64.7 (29.6-122.9)
** HPV 18**	9	2564	3.5 (1.6-6.7)	2	121	16.5 (2.0-59.7)	5	108	46.3 (15.0-108.0)
** HPV 31**	1	2625	0.4 (0.0-2.1)	0	37	0.0 (0.0-99.7)	1	37	27.0 (0.7-150.6)
** HPV 33**	6	2447	2.5 (0.9-5.3)	4	238	16.8 (4.6-43.0)	7	225	31.1 (12.5-64.1)
** HPV 35**	3	2569	1.2 (0.2-3.4)	1	86	11.6 (0.3-64.8)	4	86	46.5 (12.7-119.1)
** HPV 39**	10	2496	4.0 (1.9-7.4)	3	209	14.4 (3.0-41.9)	9	116	77.6 (35.5-147.3)
** HPV 45**	2	2552	0.8 (0.1-2.8)	1	97	10.3 (0.3-57.4)	4	72	55.6 (15.1-142.2)
** HPV 51**	8	2485	3.2 (1.4-6.3)	6	207	29.0 (10.6-63.1)	6	129	46.5 (17.1-101.2)
** HPV 52**	8	2538	3.2 (1.4-6.2)	6	264	22.7 (8.3-49.5)	8	183	43.7 (18.9-86.1)
** HPV 53**	1	2599	0.4 (0.0-2.1)	1	24	41.7 (1.1-232.2)	1	24	41.7 (1.1-232.2)
** HPV 56**	5	2472	2.0 (0.7-4.7)	4	280	14.3 (3.9-36.6)	9	249	36.1 (16.5-68.6)
** HPV 59**	6	2494	2.4 (0.9-5.2)	3	185	16.2 (3.3-47.4)	7	111	63.1 (25.4-129.9)
** HPV 66**	13	2340	5.6 (3.0-9.5)	4	430	9.3 (2.5-23.8)	13	302	43.0 (22.9-73.6)
** HPV 67**	1	2569	0.4 (0.0-2.2)	0	55	0.0 (0.0-67.1)	2	55	36.4 (4.4-131.4)
** HPV 68**	11	2386	4.6 (2.3-8.2)	5	355	14.1 (4.6-32.9)	10	190	52.6 (25.2-96.8)
** HPV 73**	0	2599	0.0 (0.0-1.4)	1	12	83.3 (2.1-464.3)	0	0	NA
** HPV 82**	1	2606	0.4 (0.0-2.1)	0	19	0.0 (0.0-194.2)	1	6	166.7 (4.2-928.6)
LR genotypes^c^									
** Any LR genotype**	51	2095	24.3 (18.1-32.0)	32	1349	23.7 (16.2-33.5)	42	857	49.0 (35.3-66.2)
** HPV 6**	28	2243	12.5 (8.3-18.0)	24	1004	23.9 (15.3-35.6)	30	646	46.4 (31.3-66.3)
** HPV 11**	9	2525	3.6 (1.6-6.8)	6	299	20.1 (7.4-43.7)	12	293	41.0 (21.2-71.5)
** HPV 40**	2	2636	0.8 (0.1-2.7)	0	48	0.0 (0.0-76.9)	2	48	41.7 (5.0-150.5)
** HPV 42**	15	2476	6.1 (3.4-10)	7	299	23.4 (9.4-48.2)	9	244	36.9 (16.9-70.0)
** HPV 54**	2	2624	0.8 (0.1-2.8)	0	0	NA	0	0	NA
** HPV 62**	3	2555	1.2 (0.2-3.4)	2	58	34.5 (4.2-124.6)	3	58	51.7 (10.7-151.2)
** HPV 72**	1	2606	0.4 (0.0-2.1)	1	7	142.9 (3.6-795.9)	0	0	NA
** HPV 81**	3	2513	1.2 (0.2-3.5)	0	86	0.0 (0.0-42.9)	3	86	34.9 (7.2-101.9)
** HPV 83**	1	2607	0.4 (0.0-2.1)	0	6	0.0 (0.0-614.8)	1	6	166.7 (4.2-928.6)
** HPV 84**	4	2569	1.6 (0.4-4.0)	0	30	0.0 (0.0-123.0)	1	30	33.3 (0.8-185.7)
Vaccine-preventable genotypes									
9V genotypes^d^	47	2248	20.9 (15.4-27.8)	44	1792	24.6 (17.8-33.0)	51	944	54.0 (40.2-71.0)
4V genotypes^e^	39	2324	16.8 (11.9-22.9)	29	1342	21.6 (14.5-31.0)	42	749	56.1 (40.4-75.8)
** HPV 16/18**	16	2447	6.5 (3.7-10.6)	5	372	13.4 (4.4-31.4)	14	247	56.7 (31.0-95.1)
** HPV 6/11**	31	2231	13.9 (9.4-19.7)	25	1065	23.5 (15.2-34.7)	33	684	48.2 (33.2-67.8)

a At least 1 of 37 HPV genotypes were detected;

b HR genotypes include HPV 16, 18, 26, 31, 33, 35, 39, 45, 51, 52, 53, 56, 59, 66, 67, 68, 69, 73, 82 (MM4), but genotype 26 and 69 in this sample were not detected;

c LR genotypes include HPV 6, 11, 40, 42, 54, 55, 61, 62, 64, 70, 71 (CP8061), 72, 81 (CP8304), 82V, 83 (MM7), 84 and 89, but genotype 55, 61, 64, 70, 71 (CP8061), 82V and 89 in this sample were not detected;

d 9V genotypes include HPV 6, 11, 16, 18, 31, 33, 45, 52, and 58;

e 4V genotypes include HPV 6, 11, 16, and 18. HPV, human papillomavirus; CI, confidence interval; HR, high-risk; LR, low-risk; NA, not available.

**Table 2:** Incidence/persistence/clearance of penile HPV infection among men who have sex with men.

HPV genotypes	Incidence			Persistence			Clearance		
	No.	Person Months	Rate (95%CI) per 1000 Person Months	No.	Person Months	Rate (95%CI) per 1000 Person Months	No.	Person Months	Rate (95%CI) per 1000 Person Months
Any genotype^a^	54	2018	26.8 (20.1-34.9)	19	1422	13.4 (8.0-20.9)	47	913	51.5 (37.8-68.5)
HR genotypes^b^									
** Any HR genotype**	42	2178	19.3 (13.9-26.1)	15	865	17.3 (9.7-28.6)	29	574	50.5 (33.8-72.6)
** HPV 16**	3	2599	1.2 (0.2-3.4)	0	0	NA	0	0	NA
** HPV 18**	2	2563	0.8 (0.1-2.8)	0	73	0.0 (0.0-50.5)	3	73	41.1 (8.5-120.1)
** HPV 33**	4	2514	1.6 (0.4-4.1)	1	92	10.9 (0.3-60.6)	3	67	44.8 (9.2-130.9)
** HPV 35**	1	2569	0.4 (0.0-2.2)	0	31	0.0 (0.0-119)	1	31	32.3 (0.8-179.7)
** HPV 39**	3	2623	1.1 (0.2-3.3)	1	36	27.8 (0.7-154.8)	1	6	166.7 (4.2-928.6)
** HPV 45**	3	2611	1.1 (0.2-3.4)	0	25	0.0 (0.0-147.6)	2	25	80.0 (9.7-289.0)
** HPV 51**	3	2599	1.2 (0.2-3.4)	0	36	0.0 (0.0-102.5)	1	36	27.8 (0.7-154.8)
** HPV 52**	7	2547	2.7 (1.1-5.7)	2	100	20.0 (2.4-72.2)	4	83	48.2 (13.1-123.4)
** HPV 59**	6	2530	2.4 (0.9-5.2)	1	143	7.0 (0.2-39.0)	8	143	55.9 (24.2-110.2)
** HPV 66**	13	2479	5.2 (2.8-9.0)	6	228	26.3 (9.7-57.3)	8	144	55.6 (24.0-109.5)
** HPV 68**	8	2556	3.1 (1.4-6.2)	0	165	0.0 (0.0-22.4)	7	165	42.4 (17.1-87.4)
** HPV 73**	2	2542	0.8 (0.1-2.8)	1	82	12.2 (0.3-67.9)	2	70	28.6 (3.5-103.2)
LR genotypes^c^									
** Any LR genotype**	37	2433	15.2 (10.7-21.0)	8	733	10.9 (4.7-21.5)	24	467	51.4 (32.9-76.5)
** HPV 6**	23	2532	9.1 (5.8-13.6)	4	543	7.4 (2.0-18.9)	19	380	50.0 (30.1-78.1)
** HPV 11**	7	2564	2.7 (1.1-5.6)	0	126	0.0 (0.0-29.3)	6	126	47.6 (17.5-103.6)
** HPV 40**	1	2611	0.4 (0.0-2.1)	2	50	40.0 (4.8-144.5)	1	38	26.3 (0.7-146.6)
** HPV 42**	4	2599	1.5 (0.4-3.9)	0	36	0.0 (0.0-102.5)	1	36	27.8 (0.7-154.8)
** HPV 54**	0	2562	0.0 (0.0-1.4)	0	38	0.0 (0.0-97.1)	1	38	26.3 (0.7-146.6)
** HPV 62**	1	2599	0.4 (0.0-2.1)	0	0	NA	0	0	NA
** HPV 84**	6	2575	2.3 (0.9-5.1)	1	66	15.2 (0.4-84.4)	2	24	83.3 (10.1-301.0)
Vaccine-preventable genotypes									
9V genotypes^d^	35	2305	15.2 (10.6-21.1)	10	902	11.1 (5.3-20.4)	31	683	45.4 (30.8-64.4)
4V genotypes^e^	29	2490	11.6 (7.8-16.7)	5	640	7.8 (2.5-18.2)	23	464	49.6 (31.4-74.4)
** HPV 16/18**	5	2563	2.0 (0.6-4.6)	0	73	0.0 (0.0-50.5)	3	73	41.1 (8.5-120.1)
** HPV 6/11**	26	2495	10.4 (6.8-15.3)	4	604	6.6 (1.8-17.0)	22	440	50.0 (31.3-75.7)

a At least 1 of 37 HPV genotypes were detected

b HR genotypes include HPV 16, 18, 26, 31, 33, 35, 39, 45, 51, 52, 53, 56, 59, 66, 67, 68, 69, 73, and 82 (MM4), but genotype 26, 31, 53, 67, 69 and 82 in this sample were not detected;

c LR genotypes include HPV 6, 11, 40, 42, 54, 55, 61, 62, 64, 70, 71 (CP8061), 72, 81 (CP8304), 82V, 83 (MM7), 84 and 89, but genotype 55, 61, 64, 70, 71 (CP8061), 72, 81 (CP8304), 82V, 83 (MM7), and 89 in this sample were not detected;

d 9V genotypes include HPV 6, 11, 16, 18, 31, 33, 45, 52, and 58;

e 4V genotypes include HPV 6, 11, 16, and 18. HPV, human papillomavirus; CI, confidence interval; HR, high-risk; LR, low-risk; NA, not available.

Sixty-five incident infections were observed at the anus (**Table 1**). Rates of anal HPV infection incidence for any genotype, any high-risk, and any low-risk among MSM were 43.6 (95% CI: 33.7–55.6), 23.3 (17.1–31.0), and 24.3 (18.1–32.0) per 1,000 pm, respectively. Individual genotypes that caused the most anal incident infections were HPV 6 (12.5/1,000 pm), 42 (6.1/1,000 pm), 66 (5.6/1,000 pm), 68 (4.6/1,000 pm), and 39 (4.0/1,000 pm). Fifty-four incident infections were observed at the penis (**Table 2**). Rates of penile HPV infection incidence for any genotype, any high-risk, and any low-risk among MSM were 26.8 (20.1–34.9), 19.3 (13.9–26.1), and 15.2 (10.7–21.0) per 1,000 pm, respectively. Individual genotypes that caused the most penile incident infections were HPV 6 (9.1/1,000 pm), 66 (5.2/1,000 pm), 68 (3.1/1,000 pm), 52 (2.7/1,000 pm), and 11 (2.7/1,000 pm).

Fifty-eight persistent infections were observed at the anus (**Table 1**). Rates of persistence of anal HPV infection for any genotype, any high-risk, and any low-risk among MSM were 23.4 (17.7–30.2), 20.6 (14.6–28.1), and 23.7 (16.2–33.5) per 1,000 pm, respectively. Individual genotypes that caused the most persistent anal infections were HPV 72 (142.9/1,000 pm), 73 (83.3/1,000 pm), 53 (41.7/1,000 pm), 62 (34.5/1,000 pm), and 51 (29.0/1,000 pm). Nineteen persistent infections were observed at the penis (**Table 2**). Rates of persistence of penile HPV infection for any genotype, any high-risk, and any low-risk among MSM were 13.4 (8.0–20.9), 17.3 (9.7–28.6), and 10.9 (4.7–21.5) per 1,000 pm, respectively. Individual genotypes that caused the most penile persistent infections were HPV 40 (40.0/1,000 pm), 39 (27.8/1,000 pm), 66 (26.3/1,000 pm), 52 (20.0/1,000 pm), and 84 (15.3/1,000 pm).

Sixty-six cleared infections were observed at the anus (**Table 1**). Rates of anal HPV infection clearance for any genotype, any high-risk, and any low-risk among MSM were 58.3 (45.1–74.1), 55.0 (41.0–72.3), and 49.0 (35.3–66.2) per 1,000 pm, respectively. Individual genotypes that had the most cleared anal infections were HPV 82 (166.7/1,000 pm), 83 (166.7/1,000 pm), 39 (77.6/1,000 pm), 18 (64.7/1,000 pm), and 66 (63.1/1,000 pm). Forty-seven cleared infections were observed at the penis (**Table 2**). Rates of penile HPV infection clearance for any genotype, any high-risk, and any low-risk among MSM were 51.5 (37.8–68.5), 50.5 (33.8–72.6), and 28.6 (3.5–103.2) per 1,000 pm, respectively. Individual genotypes that had the most cleared penile infections were HPV 39 (166.7/1,000 pm), 84 (83.3/1,000 pm), 45 (80.0/1,000 pm), 59 (55.9/1,000 pm), and 66 (55.6/1,000 pm).

### Correlates of HPV incidence and clearance among MSM

In the multivariable model, men who did not always use condoms in receptive sex (AOR 2.06, 95% CIs 1.14–3.72) were more likely to acquire anal HPV infection of any genotype than those who did not have receptive sex in the past year (**Table 3**). Age at recruitment (1.05, 1.01–1.09) was positively associated with penile HPV incidence of any genotype. MSM with more than one sex partner in receptive anal sex (0.53, 0.30–0.94) were less likely to clear any HPV infection at the anus (**Table 4**). MSM who were unemployed/students (0.55, 0.30–0.98) were less likely to clear any HPV infection at the penis.

**Table 3:** Factors associated with the incidence of any HPV genotype among men who have sex with men.

Variables^a^	OR (95%CI)	*P*	AOR (95%CI)	*P*
Anus (N=201)				
Age at recruitment (years)	–	–	1.01 (0.97-1.05)	0.547
Condom use in receptive anal sex in the past year^b^				
No receptive anal sex	ref		ref	
Always	1.32 (0.71-2.44)	0.385	1.35 (0.72-2.51)	0.348
Not always	1.96 (1.12-3.42)	0.018	2.06 (1.14-3.72)	**0.017**
Refuse to answer	1.09 (0.46-2.55)	0.848	1.11 (0.47-2.63)	0.807
Penis (N=201)				
Age at recruitment (years)	–	–	1.05 (1.01-1.09)	**0.006**
Sexual orientation				
Homosexuality	ref	–	ref	–
Others	1.62 (0.90-2.90)	0.107	1.67 (0.95-2.96)	0.076
No. of partner in insertive anal sex in the past year^c^				
0	ref	–	ref	–
1	0.57 (0.18-1.79)	0.334	0.57 (0.17-1.93)	0.368
≥2	0.88 (0.29-2.70)	0.822	0.90 (0.28-2.94)	0.863
Refuse to answer	1.01 (0.30-3.41)	0.991	0.95 (0.25-3.64)	0.940
Condom use in insertive anal sex in the past year^c^				
No insertive anal sex	ref	–	ref	–
Always	2.18 (0.59-8.05)	0.241	2.19 (0.56-8.64)	0.262
Not always	2.25 (0.66-7.64)	0.192	2.56 (0.69-9.44)	0.158
Refuse to answer	1.37 (0.31-5.96)	0.675	1.46 (0.30-7.02)	0.634
STIs diagnosis in lifetime				
No	ref	–	ref	–
Yes	1.14 (0.45-2.89)	0.780	0.98 (0.41-2.34)	0.966
Refuse to answer	1.07 (0.32-3.66)	0.908	0.95 (0.28-3.28)	0.937
STIs diagnosis in past year				
No	ref	–	ref	–
Yes	1.45 (0.50-4.18)	0.489	1.85 (0.67-5.13)	0.235
Refuse to answer	1.45 (0.39-5.42)	0.578	1.64 (0.45-6.01)	0.458

a Variables with a P < 0.2 in univariate analyses, entered into the multivariable models;

b Partner's penis in participant's anus;

c Participant's penis in partner's anus. OR, odds ratios; CI, confidence interval; P, p-value; AOR, adjusted odds ratios; STIs, sexually transmitted infections.The meaning of the bold values are statistically significant (P<0.05).

**Table 4:** Factors associated with the clearance of any HPV genotype among men who have sex with men.

Variables^a^	OR (95%CI)	*P*	AOR (95%CI)	*P*
Anus (N=106)				
Age at recruitment (years)	–	–	1.00 (0.03-2.40)	0.980
Education				
High school and below	ref	–	ref	–
College/University and above	1.53 (0.64-3.67)	0.338	1.53 (0.64-3.69)	0.342
Monthly income (NT$^b^)				
<20000	ref	–	ref	–
[20000-40000)	1.33 (0.80-2.22)	0.278	1.33 (0.79-2.25)	0.287
≥40000	1.56 (0.79-3.07)	0.196	1.57 (0.81-3.02)	0.178
Circumcised				
Yes	ref	–	ref	–
No/Unknown^c^	0.66 (0.38-1.15)	0.142	0.66 (0.36-1.19)	0.166
No. of partner in receptive anal sex in the past year^d^				
0	ref	–	ref	–
1	1.14 (0.56-2.33)	0.719	1.14 (0.53-2.46)	0.741
≥2	0.53 (0.30-0.92)	0.023	0.53 (0.30-0.94)	**0.029**
Refuse to answer	0.95 (0.48-1.87)	0.883	0.95 (0.47-1.93)	0.885
Penis (N=61)				
Age at recruitment (years)	–	–	0.99 (0.94-1.04)	0.644
Employment				
Employed	ref	–	ref	–
Unemployed/student	0.57 (0.33-0.99)	0.047	0.55 (0.30-0.98)	**0.044**
No. of partner in insertive anal sex in the past year^e^				
0	ref	–	ref	–
1	0.92 (0.32-2.65)	0.881	0.92 (0.32-2.68)	0.885
≥2	2.00 (0.75-5.34)	0.166	2.03 (0.75-5.50)	0.163
Refuse to answer	1.32 (0.46-3.79)	0.601	1.38 (0.47-4.01)	0.557
Condom use in insertive anal sex in the past year^e^				
No insertive anal sex	ref	–	ref	–
Always	1.17 (0.34-3.97)	0.801	1.16 (0.34-3.96)	0.818
Not always	1.09 (0.36-3.28)	0.879	1.04 (0.34-3.17)	0.941
Refuse to answer	1.01 (0.28-3.63)	0.983	1.01 (0.28-3.63)	0.988

a Factors with a P < 0.2 in univariate analyses, entered into the multivariable models;

b 1 US$ = 31.7 NT$ in October 2022;

c Two individuals did not report circumcision information;

d Partner's penis in participant's anus;

e Participant's penis in partner's anus. OR, odds ratios; CI, confidence interval; P, p-value; AOR, adjusted odds ratios; STIs, sexually transmitted infections.The meaning of the bold values are statistically significant (P<0.05).

**Figure 1:**
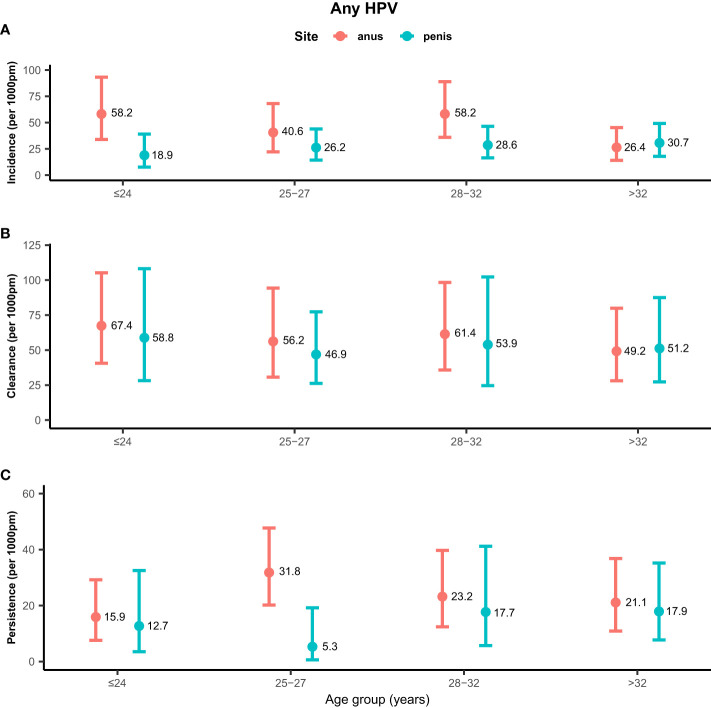
Distribution of any genotype HPV incidence/clearance/persistence rates among men who have sex with men by age group. **(A)** Incidence rate for any genotype. **(B)** Clearance rate for any genotype. **(C)** Persistence rate for any genotype. HPV, human papillomavirus; Any, any genotype.

**Figure 2:**
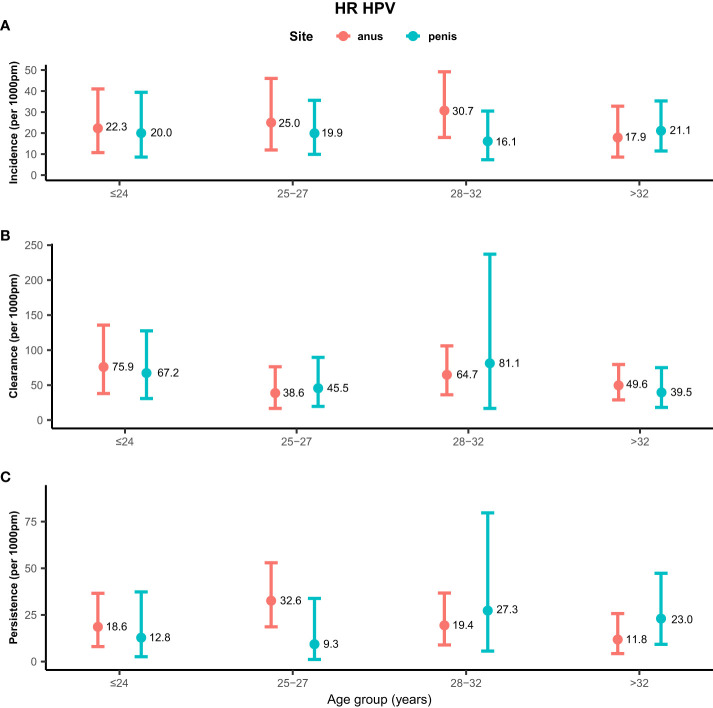
Distribution of high-risk genotype HPV incidence/clearance/persistence rates among men who have sex with men by age group. **(A)** Incidence rate for the HR genotype. **(B)** Clearance rate for the HR genotype. **(C)** Persistence rate for the HR genotype. HPV, human papillomavirus; HR, high-risk genotype.

Rates of any genotype HPV incidence, clearance, and persistence across age groups are shown in **Figure 1**. MSM aged ≤24 years (58.2/1,000 pm) and 28–32 years (58.2/1,000 pm) had the highest incidence rate of any genotype HPV at the anus, while men aged >32 years (26.4/1,000 pm) had the lowest rate. Inconsistent results were found for incidence rates of penile HPV infection, with the highest rate in men aged >32 years (30.7/1,000 pm) and the lowest rate in men aged ≤ 24 years (18.9/1,000 pm). The clearance of any genotype HPV infections was highest in men aged ≤24 years at the anus (67.4/1,000 pm) and penis (58.8/1,000 pm). Men aged 25–27 years had a high persistence rate of any genotype HPV infection at the anus (31.8/1,000 pm), but had a low persistence rate at the penis (5.3/1,000 pm). Persistence of anal HPV infection was lowest in men aged ≤24 years (15.9/1,000 pm). Rates of high-risk genotype HPV incidence, clearance, and persistence across age groups are shown in **Figure 2**. The incidence of high-risk genotype HPV infection at the anus was highest in men aged 28–32 years (30.7/1,000 pm). Men aged 25–27 years (38.6/1,000 pm) had the lowest clearance rate of high-risk HPV infection at the anus and men aged >32 years (39.5/1,000 pm) had the lowest rate at the penis. The lowest persistence rates of high-risk HPV at the anus and penis were 11.8/1,000 pm (>32 years) and 9.3/1,000 pm (25-27 years), respectively.

## Discussion

This cohort study of 201 MSM attending community health centers is one of the few studies investigating the incidence, persistence, and clearance of anogenital HPV among MSM in Asia. Most of the participants were young, well-educated, and employed. We found high rates of incident HPV at the anus and penis in this population, particularly infection with the HPV 6 genotype. Persistent events of 9V genotypes were common. Approximately one-third of MSM had incident infection of any anal HPV at follow-up visits in our study. Incidence rates of penile HPV were higher among older MSM than younger MSM.

Owing to the distinct epidemiological assumptions of incidence, persistence, and clearance rates for grouped HPV types between different studies, these data are not comparable (24, 25). Here, we only discuss the performances of individual and grouped genotypes and potential mechanisms in this study and others. High-risk HPV genotypes have been demonstrated to be the carcinogenic factors of various cancers (26). The incidence rates for HPV 6 and 8 among high-risk genotypes were similar to a previous study in Asia (27). Consistent with a study in Xinjiang, China (11), we found that HPV 39 had a relatively high incidence rate and HPV 31 had a relatively low clearance rate at the anus. We found that the incidence rates of anogenital HPV infections were high for HPV 66 (anal, 5.6/1,000 pm; penile, 5.2/1000 pm) and HPV 68 (anal, 4.6/1,000 pm; penile, 3.1/1,000 pm) among all high-risk genotypes in MSM. Meanwhile, there were high persistence rates at the anus and penis for HPV 51 (29.0/1,000 pm) and HPV 66 (26.3/1,000 pm), respectively. Such a shifting distribution of non-vaccine HPV high-risk genotypes has been observed in females by Fischer et al. (2016), particularly HPV 51, 53, 56, and 66 (28). Thus, protected measures beyond HPV vaccination, such as condom use and STI screening, should be strengthened, especially in resource-limited countries.

Low-risk HPV genotypes have been proven to be attributed to genital warts and other lesions (26). High incidence and persistence rates were observed for anal HPV 6 and 42 among low-risk genotypes in this study, which are similar to the patterns found in a previous study (12). The highest persistence rate of HPV 6 in a large cohort study in Liuzhou, China was among low-risk genotypes (29, 30). This body of evidence suggests that HPV 6 is probably the most prevalent low-risk genotype in China. We found that the clearance rates of penile HPV infections for HPV 6 and 11 were relatively high compared with other genotypes, and HPV 42 was difficult to clear at both the anus and penis, which is in line with a French study that reported the clearance rates of HPV 6 (penile, 160.2/1,000 pm), 11 (penile, 79.1/1,000 pm), and 42 (anal, 3.0/1,000 pm; penile, 70.5/1,000 pm) (16). Nevertheless, the clearance rate of HPV 42 at the anus was highest among the low-risk genotypes in the Xinjiang study (11), and was relatively high in another study in mainland China (12). Although genital warts are not fatal, prevention of low-risk HPV infections and subsequent lesions is vital for MSM. The preventive effect of the 4V vaccine was predominately driven by the decrease in HPV 6 and 11 among MSM, according to a clinical trial in Australia (31).

For anal HPV infection, the inconsistent use of condoms was positively associated with any HPV incident in our analyses. Zhang et al. (2022) found that the absence of the latest anal sex condom use (HR, 1.80; 95% CI 1.10, 2.94) could be a potential risk factor (11). Additionally, Marra et al. reported inconsistent condom use (1.94; 1.03, 3.67), which is a risk factor for HPV 16 infection. In this study, MSM who had at least two receptive anal sex partners in the past year were less likely to clear anal HPV infection than men who had no sex partners. Multiple sexual partners may be an independent risk factor for anal HPV infection. Nyitray et al. (2016) found that MSM who had at least two anal sex partners in the past 3 months were more likely to have high persistence rates for high-risk HPV genotype than men who had less than two partners (32). For penile HPV infection, the incidence rate increased with age in the multivariable model. A national survey conducted in the US found that the weighted prevalence of penile HPV infection among male respondents increased with age (33). Older men could be more vulnerable to penile HPV infection due to the decreased capability of virus clearance and the rising latent period of HPV infection (34).

This community cohort study has some limitations. First, the sample size recruited for our study was limited in this region. The participants of the study were mostly young men; therefore, the results could not generalize to MSM of all ages. Additionally, the participants who were recruited from the community health center in the city could not represent general MSM, particularly rural males. Future studies on MSM populations could try to include larger sample sizes to enhance the understanding of HPV natural history in this population. In addition, we did not use a more rigorous definition of the epidemiological rates (e.g., one negative visit followed by two consecutive positive visits for incidence, and two consecutive positive visits followed by one negative visit for clearance) due to the limited effective follow-up (35). Thus, it is inevitable that an HPV transient deposition is counted as an incident infection in the analyses. Moreover, recall or social desirability biases could have occurred when the participants filled out the questionnaire about their sexual behaviors, which can impede the validity of data. The results of the study could be hampered by uncontrolled confounding bias as it is an observational study. Furthermore, test results based on limited follow-up visits do not truly reflect the dynamic infection/clearance of HPV, which is likely to be influenced by the incubation period of the virus. This study is characterized by the undertaking of a 3-year observational cohort study among young MSM. The study was conducted in community settings, which facilitated the timely management of on-site surveys and ensured the uniformity of data collection, sampling, and testing processes.

## Conclusions

In conclusion, the high incidence and low clearance of anogenital HPV infection among MSM in this study highlight the importance of targeting this population with HPV vaccination. In light of the high burden of both vaccine and non-vaccine HPV genotypes, biochemical measures and other precautions are warranted.

## Data availability statement

The original contributions presented in the study are included in the article/**Supplementary Materials**, further inquiries can be directed to the corresponding authors.

## Ethics statement

The studies involving human participants were reviewed and approved by Ethics Committee of the National Cheng Kung University Hospital (reference number: A-BR-103-075). The patients/participants provided their written informed consent to participate in this study.

## Author contributions

HZ, CS and XZ conceived the study. CS and Y-FY did material preparation, data collection and data management. XZ, ZL, and TT conducted data cleaning and statistical analysis. XZ and TT drafted the manuscript. All authors critically revised the manuscript. All authors contributed to the article and approved the submitted version.
