# Corrigendum: Incidence, persistence, and clearance of anogenital human papillomavirus among men who have sex with men in Taiwan: a community cohort study

**DOI:** 10.3389/fimmu.2023.1244001

**Published:** 2023-07-05

**Authors:** Xinyi Zhou, Tian Tian, Zhen Lu, Yi-Fang Yu, Yuwei Li, Yiguo Zhou, Yi-Fan Lin, Carol Strong, Huachun Zou

**Affiliations:** ^1^ School of Public Health (Shenzhen), Sun Yat-sen University, Shenzhen, China; ^2^ Department of Public Health, College of Medicine, National Cheng Kung University, Tainan, Taiwan; ^3^ School of Public Health, Peking University, Beijing, China; ^4^ Kirby Institute, University of New South Wales, Sydney, Australia

**Keywords:** human papillomavirus, incidence, persistence, clearance, men who have men with men

**Incorrect Author List**

In the published article, the author list was incorrectly written as Xinyi Zhou^1^, Tian Tian^1^, Zhen Lu^1^, Yi-Fang Yu^2^, Yuwei Li^1^, Yiguo Zhou^3^, Yi-Fan Lin^1^, Carol Strong^2*^, Huachun Zou ^1,4*^.

The correct version is Xinyi Zhou^1†^, Tian Tian^1†^, Zhen Lu^1†^, Yi-Fang Yu^2^, Yuwei Li^1^, Yiguo Zhou^3^, Yi-Fan Lin^1^, Carol Strong^2*^, Huachun Zou^1,4*^

The authors apologize for this error and state that this does not change the scientific conclusions of the article in any way. The original article has been updated.

