# Supplementary material for: Incidence, persistence, and clearance of anogenital human papillomavirus among men who have sex with men in Taiwan: a community cohort study
Source: Front Immunol. 2023 Jun 20;14:1190007. doi: 10.3389/fimmu.2023.1190007 (PMC10318129; doi:10.3389/fimmu.2023.1190007)
Supplement: Supplementary file 1 [file Table_1.docx]

**Table S1. Demographic characteristics and sexual behaviors among men who have sex with men.**

|  | **Retained in cohort**  **(N = 201)** | | **Lost in follow-up**  **(N = 50)** | | **Total**  **(N=251)** |
| --- | --- | --- | --- | --- | --- |
|  | **No.** | **%** | **No.** | **%** | **%^a^** |
| **Age at recruitment (years) [Median (IQR)]** | 27 (24-32) |  | 27 (24-33) |  |  |
| <20 | 1 | 0.4 | 1 | 0.4 | 0.8 |
| 20-30 | 126 | 50.2 | 32 | 12.7 | 62.9 |
| 30-40 | 58 | 23.1 | 13 | 5.2 | 28.3 |
| >40 | 16 | 6.4 | 4 | 1.6 | 8.0 |
| **Marriage** |  |  |  |  |  |
| Single/single but dating | 114 | 45.4 | 27 | 10.8 | 56.2 |
| In a committed relationship | 87 | 34.7 | 23 | 9.2 | 43.8 |
| **Education** |  |  |  |  |  |
| High school and below | 18 | 7.2 | 8 | 3.2 | 10.4 |
| College/University and above | 183 | 72.9 | 42 | 16.7 | 89.6 |
| **Employment** |  |  |  |  |  |
| Employed | 129 | 51.4 | 35 | 13.9 | 65.3 |
| Unemployed/student | 72 | 28.7 | 15 | 6.0 | 34.7 |
| **Monthly income (NT$^b^)** |  |  |  |  |  |
| <20000 | 70 | 28.0 | 14 | 5.6 | 33.6 |
| [20000-40000) | 82 | 32.8 | 22 | 8.8 | 41.6 |
| ≥40000 | 49 | 19.6 | 13 | 5.2 | 24.8 |
| **Sexual orientation** |  |  |  |  |  |
| Homosexuality | 160 | 63.7 | 43 | 17.1 | 80.8 |
| Others | 41 | 16.3 | 7 | 2.8 | 19.2 |
| **Circumcised** |  |  |  |  |  |
| Yes | 46 | 18.3 | 18 | 7.2 | 25.5 |
| No/Unknown^c^ | 155 | 61.8 | 32 | 12.7 | 74.5 |
| **HPV testing in life time** |  |  |  |  |  |
| Yes | 18 | 7.2 | 2 | 0.8 | 8.0 |
| No | 183 | 72.9 | 48 | 19.1 | 92.0 |
| **HPV vaccine uptake** |  |  |  |  |  |
| Yes | 7 | 2.8 | 5 | 2.0 | 4.8 |
| No | 194 | 77.6 | 44 | 17.6 | 95.2 |
| **No. of partner in insertive anal sex in the past year^d^*** |  |  |  |  |  |
| 0 | 56 | 22.3 | 9 | 3.6 | 25.9 |
| 1 | 50 | 19.9 | 6 | 2.4 | 22.3 |
| ≥2 | 59 | 23.5 | 18 | 7.2 | 30.7 |
| Refuse to answer | 36 | 14.3 | 17 | 6.8 | 21.1 |
| **No. of partner in receptive anal sex in the past year^e^** |  |  |  |  |  |
| 0 | 55 | 21.9 | 17 | 6.8 | 28.7 |
| 1 | 41 | 16.3 | 6 | 2.4 | 18.7 |
| ≥2 | 73 | 29.1 | 12 | 4.8 | 33.9 |
| Refuse to answer | 32 | 12.7 | 15 | 6.0 | 18.7 |
| **Condom use in insertive anal sex in the past year^d^** |  |  |  |  |  |
| No insertive anal sex | 49 | 19.5 | 9 | 3.6 | 23.1 |
| Always | 53 | 21.1 | 13 | 5.2 | 26.3 |
| Not always | 70 | 27.9 | 20 | 8.0 | 35.9 |
| Refuse to answer | 29 | 11.6 | 8 | 3.2 | 14.7 |
| **Condom use in receptive anal sex in the past year^e^** |  |  |  |  |  |
| No insertive anal sex | 54 | 21.6 | 16 | 6.4 | 28.0 |
| Always | 63 | 25.2 | 10 | 4.0 | 29.2 |
| Not always | 59 | 23.6 | 16 | 6.4 | 30.0 |
| Refuse to answer | 24 | 9.6 | 8 | 3.2 | 12.8 |
| **STIs diagnosis in lifetime** |  |  |  |  |  |
| No | 137 | 54.6 | 32 | 12.7 | 67.3 |
| Yes | 52 | 20.7 | 16 | 6.4 | 27.1 |
| Refuse to answer | 12 | 4.8 | 2 | 0.8 | 5.6 |
| **STIs diagnosis in the past year** |  |  |  |  |  |
| No | 160 | 63.7 | 37 | 14.7 | 78.4 |
| Yes | 31 | 12.4 | 9 | 3.6 | 16.0 |
| Refuse to answer | 10 | 4.0 | 4 | 1.6 | 5.6 |

**P*<0.05 (variables with differences: “No. of partner in insertive anal sex in the past year”, *P*=0.021). ^a^ Sum of the proportions at the baseline; ^b^ 1 US$ = 31.7 NT$ in October 2022; ^c^ Two individuals did not report circumcision information; ^d^ Participant's penis in partner's anus; ^e^ Partner's penis in participant's anus. IQR, interquartile range; NT, New Taiwan Dollar; HPV, human papillomavirus; STIs, sexually transmitted infections.

**Table S2. Attrition analyses of HPV incidence rates at the follow up.**

| **Site** | **Type** | **Group^a^** | **Incident events** | **Person-months (pm)** | **Incidence rate (/1000 pm)** | ***P*** |
| --- | --- | --- | --- | --- | --- | --- |
| Anal | Any | A | 31 | 787 | 39.3 | 0.964 |
|  |  | B | 27 | 655 | 41.2 |  |
|  | HR | A | 23 | 882 | 26.1 | 0.982 |
|  |  | B | 20 | 728 | 27.5 |  |
|  | LR | A | 18 | 934 | 19.3 | 0.985 |
|  |  | B | 16 | 779 | 20.5 |  |
| Penile | Any | A | 25 | 992 | 25.2 | 1.000 |
|  |  | B | 21 | 805 | 26.1 |  |
|  | HR | A | 15 | 1035 | 14.5 | 0.980 |
|  |  | B | 13 | 829 | 15.7 |  |
|  | LR | A | 13 | 1055 | 12.3 | 1.000 |
|  |  | B | 11 | 868 | 12.7 |  |

^a^ Group: A, MSM who participated in the 6-month follow up (N=182); B, MSM who retained in the 12-month follow up (N=148). HPV, human papillomavirus; Any, any genotype; HR, high-risk genotype; LR, low-risk genotype; *P*, p-value.
